# Replication of newly proposed TNM staging system for medullary thyroid carcinoma: a nationwide study

**DOI:** 10.1530/EC-18-0494

**Published:** 2018-12-14

**Authors:** Jes Sloth Mathiesen, Jens Peter Kroustrup, Peter Vestergaard, Per Løgstrup Poulsen, Åse Krogh Rasmussen, Ulla Feldt-Rasmussen, Sten Schytte, Stefano Christian Londero, Henrik Baymler Pedersen, Christoffer Holst Hahn, Jens Bentzen, Sören Möller, Mette Gaustadnes, Maria Rossing, Finn Cilius Nielsen, Kim Brixen, Christian Godballe, Danish Thyroid Cancer Group (DATHYRCA)

**Affiliations:** 1 Department of ORL Head & Neck Surgery and Audiology, Odense University Hospital, Odense, Denmark; 2 Department of Clinical Research, University of Southern Denmark, Odense, Denmark; 3 Department of Clinical Medicine and Endocrinology, Aalborg University Hospital, Aalborg, Denmark; 4 Steno Diabetes Center North Jutland, Aalborg, Denmark; 5 Department of Internal Medicine and Endocrinology, Aarhus University Hospital, Aarhus, Denmark; 6 Department of Medical Endocrinology, Copenhagen University Hospital, Copenhagen, Denmark; 7 Department of ORL Head & Neck Surgery, Aarhus University Hospital, Aarhus, Denmark; 8 Department of ORL Head & Neck Surgery, Aalborg University Hospital, Aalborg, Denmark; 9 Department of ORL Head & Neck Surgery, Copenhagen University Hospital, Copenhagen, Denmark; 10 Department of Oncology, Herlev Hospital, Herlev, Denmark; 11 Odense Patient data Explorative Network (OPEN), Odense University Hospital, Odense, Denmark; 12 Department of Molecular Medicine, Aarhus University Hospital, Aarhus, Denmark; 13 Center for Genomic Medicine, Copenhagen University Hospital, Copenhagen, Denmark

**Keywords:** thyroid, endocrine cancers, rare diseases/syndromes

## Abstract

A recent study proposed new TNM groupings for better survival discrimination among stage groups for medullary thyroid carcinoma (MTC) and validated these groupings in a population-based cohort in the United States. However, it is unknown how well the groupings perform in populations outside the United States. Consequently, we conducted the first population-based study aiming to evaluate if the recently proposed TNM groupings provide better survival discrimination than the current American Joint Committee on Cancer (AJCC) TNM staging system (seventh and eighth edition) in a nationwide MTC cohort outside the United States. This retrospective cohort study included 191 patients identified from the nationwide Danish MTC cohort between 1997 and 2014. In multivariate analysis, hazard ratios for overall survival under the current AJCC TNM staging system vs the proposed TNM groupings with stage I as reference were 1.32 (95% CI: 0.38–4.57) vs 3.04 (95% CI: 1.38–6.67) for stage II, 2.06 (95% CI: 0.45–9.39) vs 3.59 (95% CI: 1.61–8.03) for stage III and 5.87 (95% CI: 2.02–17.01) vs 59.26 (20.53–171.02) for stage IV. The newly proposed TNM groupings appear to provide better survival discrimination in the nationwide Danish MTC cohort than the current AJCC TNM staging. Adaption of the proposed TNM groupings by the current AJCC TNM staging system may potentially improve accurateness in survival discrimination. However, before such an adaption further population-based studies securing external validity are needed.

## Introduction

Medullary thyroid carcinoma (MTC) is a rare neuroendocrine tumor with an incidence of 0.19 per 100,000 per year and a prevalence of 3.8 per 100,000 inhabitants. MTC is divided into a sporadic and hereditary type accounting for approximately 75 and 25%, respectively (1).

MTC can display a highly variable biological behavior, ranging from indolent to very aggressive (2, 3, 4). This necessitates strong outcome predictors, for example, the American Joint Committee on Cancer (AJCC) TNM staging system (5, 6, 7). The current AJCC TNM version (seventh and eighth edition) by large mirrors that for papillary and follicular thyroid carcinoma in spite of the fact that MTC differs considerably from the other histological subtypes (2). For this and other reasons several studies have questioned the accuracy of the AJCC TNM staging system for MTC and thus proposed different modifications (3, 8, 9, 10).

Based on data from the US National Cancer Database (11), a large population-based study recently proposed new TNM groupings for better discrimination of mortality risk among the stage groups (2). Application of the proposed TNM groupings to a data set from the Surveillance, Epidemiology, and End Results Program (12) demonstrated a better stage separation compared to that of the current AJCC TNM staging system (2). In brief, overall differences between the current AJCC TNM staging system and the newly proposed groupings include categorization of patients with small tumors and local metastases in stages I–II and inclusion of only patients with distant metastases in stage IV in the proposed TNM groupings. It is however unknown how well these groupings perform in populations outside the United States.

Consequently, we conducted the first population-based study aiming to evaluate if the recently proposed TNM groupings provide better survival discrimination than the current AJCC TNM staging system in a nationwide MTC cohort outside the United States.

## Patients and methods

### Patients

This retrospective cohort study included 191 unique patients diagnosed with MTC in Denmark between January 1, 1997, and December 31, 2014.

An MTC cohort, initially comprising 476 patients diagnosed with MTC in Denmark between January 1960 and December 2014, was constructed through three nationwide registries: the Danish Thyroid Cancer Database, the Danish Cancer Registry and the Danish Pathology Register (13, 14, 15). This has been described in detail previously (1, 16). The Danish MTC cohort is subdivided by year of diagnosis into an uncertain period (1960–1996) where complete coverage could not be guaranteed and into a nationwide period (1997–2014) where coverage of the entire country was considered complete. For the purpose of this study, we extracted the 224 patients diagnosed in the nationwide period. Of the 224 patients, four were excluded as they were diagnosed by autopsy. Furthermore, for the best possible replication, our cohort was trimmed according to the cohort from the study proposing new TNM groupings (2). We therefore additionally excluded 29 patients: those <18 years at diagnosis (*n* = 10) (16, 17, 18, 19, 20), those with insufficient TNM data (*n* = 2) and those who underwent less surgery than hemithyroidectomy (*n* = 17). This resulted in 191 patients with histologically verified MTC.

The investigation was approved by the Danish Health Authority (3-3013-395/3) and the Danish Data Protection Agency (18/17801). Once approved by the former, patient consent is not necessary according to the Danish legislation.

### Methods

Data were provided by the Danish Thyroid Cancer Database (13). Where this was insufficient, data were drawn from the Danish Cancer Registry (14), the Danish Pathology Register (15) or medical records.

Covariates were age, sex, year of diagnosis and TNM stage. Also patients were classified according to MTC type as sporadic or hereditary. This was primarily based on the absence or presence of rearranged during transfection (*RET*) germline mutations. *RET* testing and MTC classification has been described elsewhere (1, 21). Staging was performed according to the current AJCC TNM staging system (seventh and eighth edition) (22, 23) and according to the newly proposed TNM groupings (2) (Table 1). Staging was based on clinical and pathological assessment. If there was a discrepancy, pathological staging overruled clinical.

**Table 1:** Distribution of 191 patients with medullary thyroid carcinoma in Denmark 1997–2014 according to the current^a^ and proposed TNM staging system^b^.

Stage	Current	*n* (%)	Proposed	*n* (%)
I	T1N0M0	51 (27)	T1N0-1aM0T2N0M0	88 (46)
II	T2-3N0M0	38 (20)	T1N1bM0T2N1a-1bM0T3N0M0	54 (28)
III	T1-3N1aM0	16 (8)	T3N1a-1bM0T4N0-1bM0	40 (21)
IV	T1-3N1bM0T4N0-1bM0T1-4N0-1bM1	86 (45)	T1-4N0-1bM1	9 (5)

^a^Staging was based on the American Joint Committee on Cancer 7th and 8th edition (22, 23); ^b^staging was based on newly proposed groupings (2).

M, metastasis; N, node; T, tumor.

### Survival

Outcomes were overall and disease-specific survival. Survival time was calculated as the time from MTC diagnosis until death, emigration or last follow-up (January 1, 2018), whichever came first. For calculation of overall and disease-specific survival, all deaths and deaths due to MTC were considered as an event, respectively.

### Statistical analysis

Continuous variables are reported as median with interquartile range or mean with standard deviation depending on distribution. Survival data were analyzed by the Kaplan–Meier method. Cox proportional hazards regression model was employed for multivariate analyses. The Akaike criterion was used to estimate the relative quality of statistical models. *P* values below 0.05 were considered significant. Multiple testing was adjusted by the Bonferroni method (24). All analyses were done using Stata 15.1 (StataCorp).

## Results

A total of 191 patients were included in the study. Patient characteristics are shown in Table 2. The overall female–male ratio was 1.51 (95 CI: 1.10–1.95). In the 42 patients with hereditary MTC, the following *RET* mutations were detected: C611W (*n* = 3), C611Y (*n* = 29), C618F (*n* = 1), C618Y (*n* = 3), C620R (*n* = 2), C634R (*n* = 1), C634Y + Y791F (*n* = 1), V804M (*n* = 1) and M918T (*n* = 1). Several of these families have been reported earlier (19, 25, 26, 27, 28, 29).

**Table 2:** Characteristics of 191 patients with medullary thyroid carcinoma in Denmark 1997–2014.

Characteristics	*n* (%)
At diagnosis	
Age, mean (s.d.) (years)	53 (16)^a^
Sex	
Female	115 (60)
Male	76 (40)
MTC type	
Sporadic	149 (78)
Hereditary	42 (22)
T category	
T1	81 (42)
T2	52 (27)
T3	24 (13)
T4	34 (18)
N category	
N0	91 (48)
N1a	20 (10)
N1b	80 (42)
M category	
M0	182 (95)
M1	9 (5)
Current TNM staging^b^	
I	51 (27)
II	38 (20)
III	16 (8)
IV	86 (45)
Thyroid surgery	
Total thyroidectomy	188 (98)
Hemithyroidectomy	3 (2)

^a^Standard deviation; ^b^staging was based on the American Joint Committee on Cancer 7th and 8th edition (22, 23).

M, metastasis; MTC, medullary thyroid carcinoma; N, node; s.d., standard deviation; T, tumor.

Under the current AJCC TNM staging system 51 (27%), 38 (20%), 16 (8%) and 86 (45%) were classified in stages I, II, III and IV, respectively. Among the patients in stages I, II, III and IV, 51 (100%), 9 (24%), 2 (13%) and 9 (10%) remained in the same group under the proposed TNM groupings. The remaining patients were reclassified to lower stage groups. Thus, the distribution of patients under the proposed TNM groupings was 88 (46%) in stage I, 54 (28%) in stage II, 40 (21%) in stage III and 9 (5%) in stage IV (Table 1).

### Survival

Median follow-up time was 7.15 years (interquartile range, 0.52–20.59). At last follow-up, 59 patients had died. Of these, 37 had died from MTC, while 22 died from other causes.

**Figure 1:**
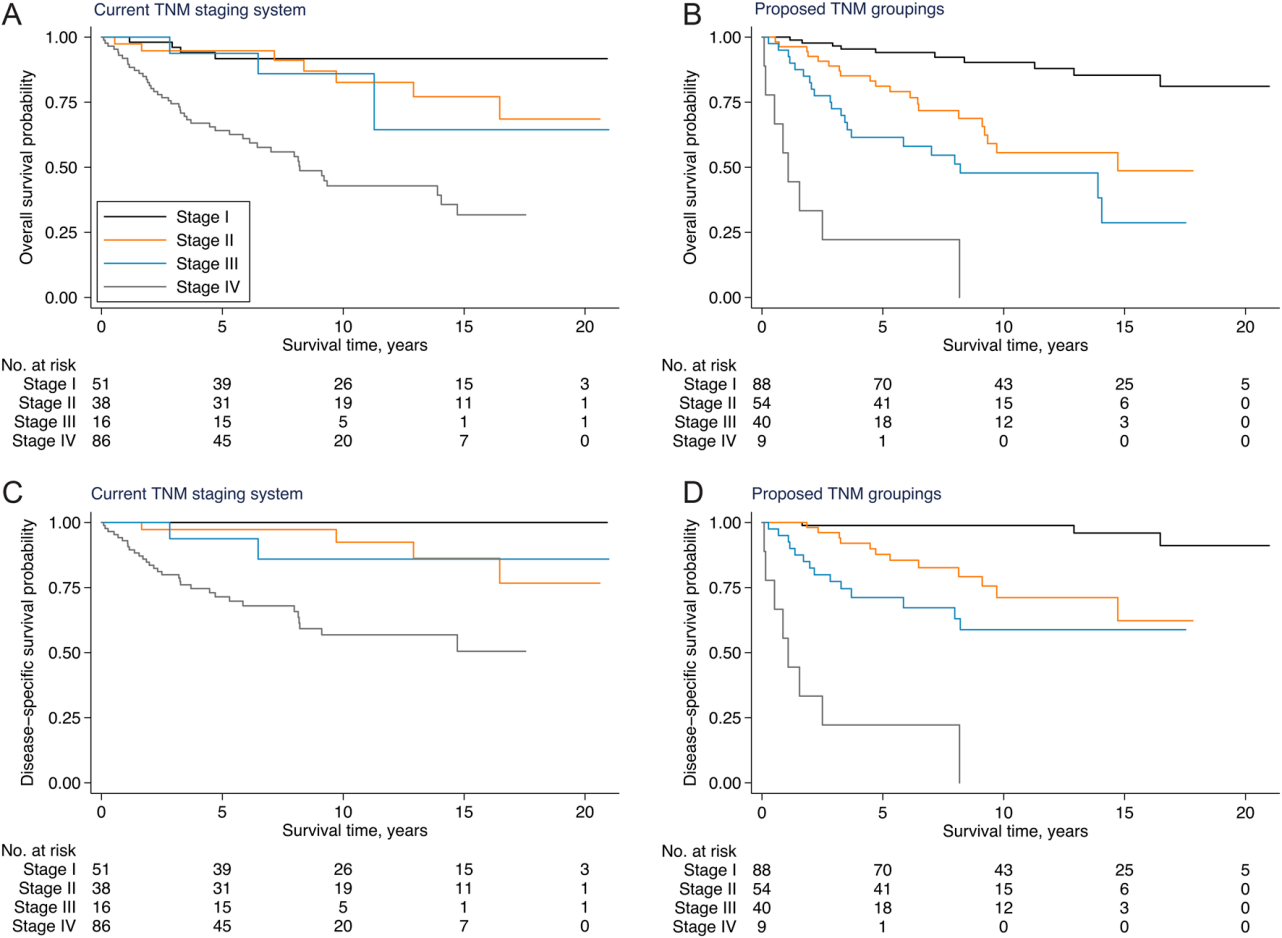
Overall and disease-specific survival in 191 patients with medullary thyroid carcinoma in Denmark 1997–2014 according to the current^a^ and proposed TNM staging system^b^. M, metastasis; N, node; T, tumor. ^a^Staging was based on the American Joint Committee on Cancer 7th and 8th edition (22, 23). ^b^Staging was based on newly proposed groupings (2).

Overall and disease-specific survival according to the current AJCC TNM staging and the proposed TNM groupings are depicted in Fig. 1 and Table 3. Hazard ratios for adjusted overall survival based on the current AJCC TNM staging and the proposed TNM groupings are presented in Table 4.

**Table 3:** Five-year survival in 191 patients with medullary thyroid carcinoma in Denmark 1997–2014 according to the current^a^ and proposed TNM staging system^b^.

Stage	Overall survival		Disease-specific survival	
	Current	Proposed	Current	Proposed
	5-year (95% CI)	5-year (95% CI)	5-year (95% CI)	5-year (95% CI)
I	92 (79–97)	94 (86–98)	100	99 (92–100)
II	95 (81–99)	81 (68–89)	97 (82–100)	88 (75–94)
III	94 (63–99)	61 (44–75)	94 (63–99)	71 (54–83)
IV	64 (53–73)	22 (3–51)	71 (60–80)	22 (3–51)

^a^Staging was based on the American Joint Committee on Cancer 7th and 8th edition (22, 23); ^b^staging was based on newly proposed groupings (2).

T, tumor; N, node; M, metastasis.

**Table 4:** Adjusted^a^ overall survival in 191 patients with medullary thyroid carcinoma in Denmark 1997–2014 according to the current^b^ and proposed TNM staging system^c^.

Stage	Current	Proposed
	HR (95% CI)	HR (95% CI)
I	1.00	1.00
II	1.32 (0.38–4.57)	3.04 (1.38–6.67)
III	2.06 (0.45–9.39)	3.59 (1.61–8.03)
IV	5.87 (2.02–17.01)	59.26 (20.53–171.02)
AIC^d^	508	489

^a^Adjusted for age, sex and year of diagnosis; ^b^staging was based on the American Joint Committee on Cancer 7th and 8th edition (22, 23); ^c^staging was based on newly proposed groupings (2); ^d^lowest AIC indicates best fitted statistical model.

AIC, Akaike information criterion; HR, hazard ratio; M, metastasis; N, node; T, tumor.

When staging was based on the current AJCC TNM staging system, adjusted overall survival did not differ significantly for stage I vs II (*P* = 0.662), stage II vs III (*P* = 0.522) or stage III vs IV (*P* = 0.082). Meanwhile, when using the proposed TNM groupings, adjusted overall survival was significantly different for stage I vs II (*P* = 0.006) and stage III vs IV (*P* < 0.001), but not stage II vs III (*P* = 0.605). Similar results were obtained after Bonferroni correction.

## Discussion

In this nationwide study, we compared the current AJCC TNM staging system to the newly proposed TNM groupings and found that the latter provides better differentiation of mortality risk for stage I vs II and stage III vs IV.

### Limitations

Our sample size is relatively small, providing lesser statistical power to detect differences in survival among the stage groups. We cannot exclude that this was the case for stage II vs III under the proposed TNM groupings. However, it was no issue in the comparison of stage I vs II and stage III vs IV, as significant differences in survival were seen in both cases, even after Bonferroni correction.

Our adjusted analysis of survival did not include the covariates: annual income, insurance and hospital type seen in the study of the proposed TNM groupings (2). However, adjustment for these covariates in a Danish setting would seem redundant, partly because medical care is free for all inhabitants, treatment of thyroid cancer is centralized to university hospitals only and partly because income and insurance in earlier multivariate analysis have been reported as non-significant predictors of overall survival (30).

For the purpose of this study, our cohort was trimmed according to the study cohort of the investigation proposing the new TNM groupings. For example, the investigation excluded patients <18 years at diagnosis from their study cohort (2) although inclusion may have been an option (31). Similarly, we excluded this patient group from our cohort. Thus, along with the other exclusion criteria, the reproducibility of the proposed TNM groupings in an unselected MTC population may be compromised. However, when reanalyzing data for the 218 patients with pertinent data from our initial cohort of 220 unselected patients, we still find a significant difference in adjusted overall survival for stage I vs II (*P* = 0.014) and stage III vs IV (*P* < 0.001) under the proposed TNM groupings.

### Survival

Our results of better survival discrimination for stage I vs II and stage III vs IV under the proposed TNM groupings are in agreement with the study proposing the new groupings (2). While better distinction between stage II and III under the proposed TNM groupings compared to the current AJCC TNM staging system may be somewhat dubious in our data set, it is indisputable for stage III vs IV. Presumably, this is explained by the fact that stage IV in the proposed TNM groupings only includes patients with distant metastases, while the current AJCC TNM staging system also includes patients with T4 or N1b disease having no evidence of distant metastases (Table 1). Thus, the current AJCC TNM staging system seems to attenuate the significance of distant metastases, despite the fact that several population-based studies report distant metastases as one of the absolute strongest prognostic indicators for survival in multivariate analysis (7, 32, 33, 34, 35, 36).

In fact, stage IV has previously consisted of patients with distant metastases only (37) as in the proposed TNM groupings (2). This was changed in 2002 in the sixth edition of the AJCC TNM staging system, where stage IV was expanded to also include patients with T4 or N1b disease regardless of distant metastases status (38). Later an institutional study, investigating the survival of 173 MTC patients according to the fifth and sixth edition of the AJCC TNM staging system found that patients with stage III and IV disease had similar disease-free survival and overall survival under the sixth edition. This led to the conclusion that the sixth edition of the AJCC TNM staging system appeared inadequate, especially for patients with stage IV disease (3) Stage IV, however, has not been changed since 2002.

In our study, the proposed TNM groupings also demonstrated better survival distinction than the current AJCC TNM staging system for stage I vs II. This may be explained by the downstaging of small tumors (T1–2) with lateral neck metastases (N1b) from stage IV in the current AJCC TNM staging system to stage II in the proposed TNM groupings (Table 1).

## Conclusion

The newly proposed TNM groupings appear to provide better survival discrimination in the nationwide Danish MTC cohort than the current AJCC TNM staging system. Adaption of the proposed TNM groupings by the current AJCC TNM staging system may potentially improve accurateness in survival discrimination. However, before such an adaption, further population-based studies securing external validity are needed.

## Declaration of interest

The authors declare that there is no conflict of interest that could be perceived as prejudicing the impartiality of the research reported.

## Funding

This work was supported by the University of Southern Denmark, the Region of Southern Denmark, Odense University Hospital, Copenhagen University Hospital, the Danish Cancer Society, the Danish Cancer Research Foundation and the A. P. Moller Foundation. The research salary of Ulla Feldt-Rasmussen is sponsored by an unrestricted research grant from the Novo Nordic Foundation.

## Author contribution statement

J S Mathiesen conceived and coordinated the study, collected data, performed data analyses and drafted the manuscript. S Möller performed data analyses and drafted the manuscript. J P Kroustrup, P Vestergaard, P L Løgstrup, Å K Rasmussen, U Feldt-Rasmuseen, S Schytte, S C Londero, H B Pedersen, C H Hahn, J Bentzen, M Gaustadnes, M Rossing, F C Nielsen, K Brixen and C Godballe participated in data collection, data analyses and drafting of the manuscript.
